# Supplementary material for: Demonstration of chip-based coupled degenerate optical parametric oscillators for realizing a nanophotonic spin-glass
Source: Nat Commun. 2020 Aug 17;11:4119. doi: 10.1038/s41467-020-17919-6 (PMC7431591; doi:10.1038/s41467-020-17919-6)
Supplement: Supplementary file 1 — Supplementary Information [file 41467_2020_17919_MOESM1_ESM.pdf]

Supplementary Information - Nanophotonic spin-glass towards coherent Ising machine

Okawachi, *et al.*

Nanophotonic spin-glass towards coherent Ising machine

Yoshitomo Okawachi[1], Mengjie Yu[1,2], Jae K. Jang[1], Xingchen Ji[3], Yun Zhao[3], Bok Young Kim[1], Michal Lipson[3] & Alexander L. Gaeta[1]
[1] *Department of Applied Physics and Applied Mathematics, Columbia University, New York, NY 10027*
[2] *School of Electrical and Computer Engineering, Cornell University, Ithaca, NY 14853 and*
[3] *Department of Electrical Engineering, Columbia University, New York, NY 10027*
(Dated: July 21, 2020)

Supplementary Note 1 - Optimization of degenerate optical parametric oscillator signal generation

Pure degenerate optical parametric oscillator (DOPO) generation in silicon nitride (SiN) microresonators requires the condition $L_{\mathrm{D}} > L_{\mathrm{NL}}$, where $L_{\mathrm{D}} = 1/\delta^2|\beta_2|$ is the dispersion length and $L_{\mathrm{NL}} = 1/2\gamma P$ is the nonlinear length [1]. Here we consider a condition where the two pumps lie in the anomalous group-velocity dispersion (GVD) regime. Supplementary Figure 1 shows the simulated GVD profile for two waveguide cross sections. 730×1050 nm corresponds to the results shown in the manuscript where the two pumps are located in the normal GVD regime. 730×1100 nm is the resonator cross section we analyze here where the two pumps are in the anomalous GVD regime.

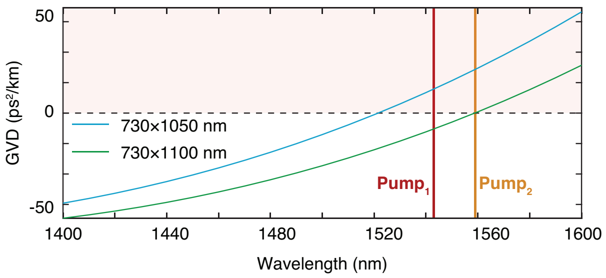

Supplementary Figure 1: **Microresonator group-velocity dispersion (GVD).** Simulated GVD of the silicon nitride (SiN) microresonator for the fundamental transverse electric (TE) mode for two different waveguide cross sections of 730×1050 nm and 730×1100 nm.

We characterize the interference between the DOPO using a setup similar to Fig. 2(c) in the main text. The SiN chip is pumped using two frequency nondegenerate pump lasers that are offset by ±1 free spectral range (FSR) from the degeneracy point. The pump wavelengths are 1546.1 nm and 1554.2 nm. One of the pumps is modulated using an acousto-optic modulator with 250-ns pulses at a repetition rate of 2 MHz. Supplementary Figure 2(a) shows the measured temporal interference signal along with each individual DOPO, and we observe constructive interference between the two signals. In addition, we observe fast modulations in the interference signal and DOPO_1. To understand the modulations, we measure the beatnote between an external continuous wave laser and two different DOPO states [Supplementary Figure 2(b)]. Modulations in the time trace are observed for an DOPO in state 1, and we observe sideband in the radio frequency (RF) spectrum that is 54 MHz from DC. We attribute these sidebands to nondegenerate frequency components that are generated in addition to the DOPO signal within a single microresonator cavity resonance. These frequency components are generated due to degenerate four-wave mixing (FWM) from a single pump from the fact that the pumps lie in the anomalous GVD regime.While it is possible to suppress the modulations via pump-cavity detuning, which can be seen in the relatively flat temporal profile for DOPO_2 which is in state 2, operation in the normal GVD regime allows for a stronger and more stable DOPO signal.

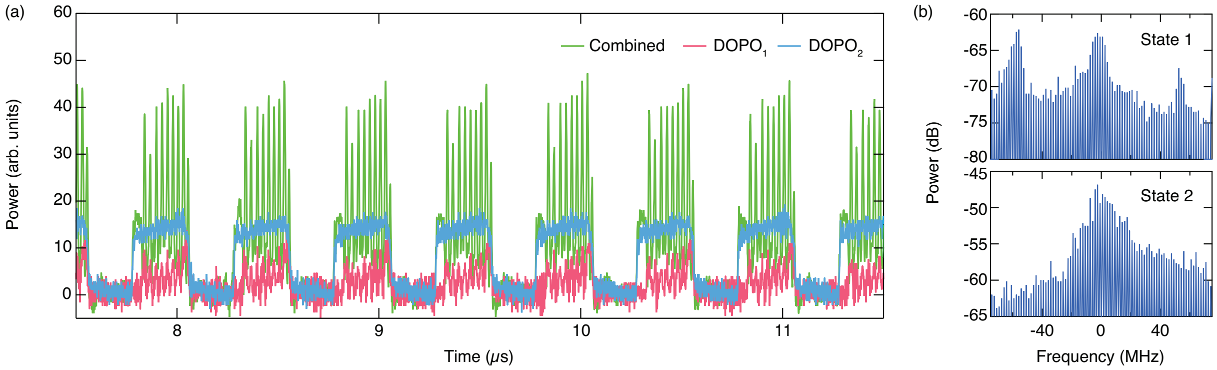

Supplementary Figure 2: **Coupled DOPO system with competing four-wave mixing (FWM) effects.** (a) Temporal interference measurement of the combined DOPO's (green) along with measurement of each DOPO. (b) RF beatnote between DOPO signal and an external cw laser for two different states. The discrete RF lines correspond to the 2 MHz modulation of the pump laser.

Supplementary Note 2 - Characterization of coupling strength

We characterize the ratio between the $DOPO_2$ power and the power that is coupled from $DOPO_1$ by measuring the power in the bus waveguide that is coupled to the microresonator corresponding to $DOPO_2$ when $DOPO_2$ is oscillating and not oscillating. The device under investigation here is the same as that from the main text. For the case where $DOPO_2$ is not oscillating, we use the integrated platinum heaters to detune the cavity resonance. Supplementary Figure 3 shows the measured optical spectra for the two cases. We observe a power difference of 26.4 dB, which corresponds to a ratio between the coupled field from $DOPO_1$ and the $DOPO_2$ field of 0.0479.

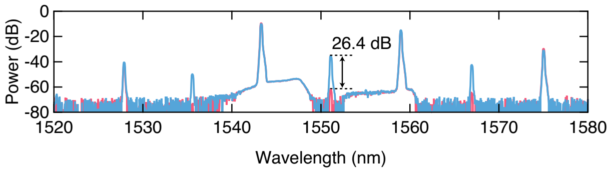

Supplementary Figure 3: **DOPO coupling ratio.** Measured output spectra from output port of $DOPO_2$ when $DOPO_2$ is (blue) and is not (magenta) oscillating. $DOPO_1$ is always oscillating for both cases. We measure the difference between the $DOPO_2$ power and the power coupled from $DOPO_1$ to be 26.4 dB.

Supplementary Note 3 - Characterization of uncoupled DOPO

We investigate the behavior of two uncoupled DOPO's generated from the same pump. The device is shown in Supplementary Figure 4. The structure is similar to that shown in Fig.2(a) in the main text but the coupling waveguide is not included here. The microresonators have an FSR of 500 GHz and a cross section of 730×1050 nm. The two pumps are coupled in and are split 50/50 using a multimode interference (MMI) splitter and sent to $DOPO_1$ (bottom) and $DOPO_2$ (top). The pumps are offset by ±2 FSR's from the degeneracy point. The experimental setup for DOPO generation and the interference measurement is the same as that shown in Supplementary Figure 2(c). The high wavelength pump is modulated using an acousto-optic modulator with 310-ns pulses at a repetition rate of 400 kHz in order to ensure that the DOPO is extinguished after each generation such that each successive DOPO initiation is uncorrelated. Supplementary Figure 5 shows the generated spectrum for $DOPO_1$ and $DOPO_2$.

Supplementary Figure 6 shows the time domain interference measurement (green) along with the temporal

measurement of each individual DOPO signal (red and blue). In contrast to the coupled DOPO measurement shown in Fig. 4 in the main text, we observe that there is no fixed phase relationship between the two generated DOPO's indicating that each of the OPO's are oscillating independently and generating uncorrelated bi-phase states.

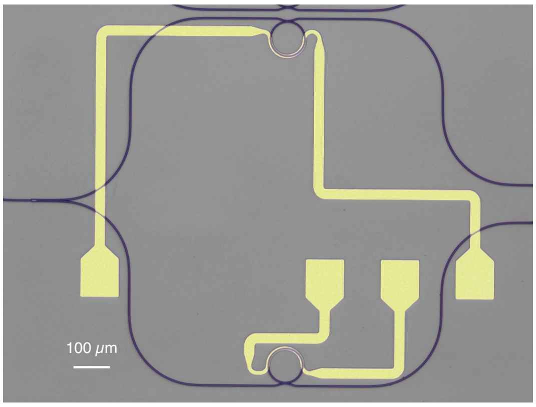

Supplementary Figure 4: **Microscope image of uncoupled DOPO device.** An multi-mode inteference (MMI) splitter is used to route the pumps to $DOPO_1$ (bottom) and $DOPO_2$ (top). Contrary to the device shown in Fig. 2(b) in the main text, the coupling waveguide is omitted.

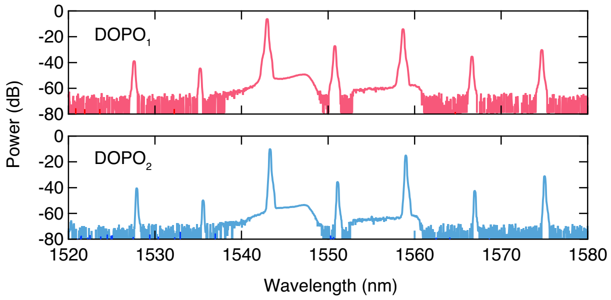

Supplementary Figure 5: **Optical spectra of two uncoupled DOPO signals.** The measured spectra of the primary DOPO (top, $DOPO_1$) and the secondary DOPO (bottom, $DOPO_2$).

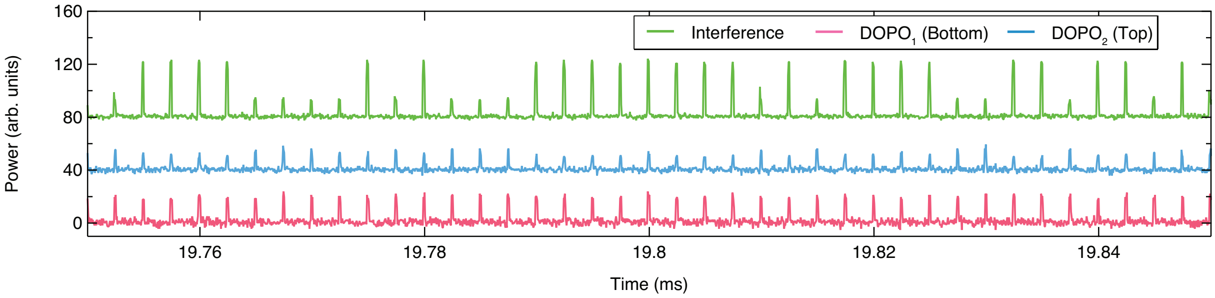

Supplementary Figure 6: **Phase characterization of uncoupled DOPO's.** Temporal interference measurement of the combined DOPO's (green) along with measurement of $DOPO_1$ (red) and $DOPO_2$ (blue) for uncoupled DOPO system. For clarity, $DOPO_2$ and the interference are offset from $DOPO_1$ in power by 40 and 80, respectively.

Supplementary Note 4 - Theoretical modeling of coupled DOPO system

The propagation of a slowly varying light field envelope $B^{(m)}(z,t)$ in a one-dimensional dispersive medium exhibiting instantaneous Kerr nonlinearity is described by a generalized nonlinear Schrödinger equation which can be expressed in the frequency domain as

$$\frac{\partial \tilde{B}^{(m)}}{\partial z} = \bigg(-\frac{\alpha_0}{2} + i\beta_1\Omega + i\sum_{k\geq 2}\frac{\beta_k}{k!}\Omega^k \bigg)\tilde{B}^{(m)}(z,\Omega) + i\gamma\big[\bar{\tilde{B}}^{(m)} \circledast \big(\tilde{B}^{(m)} \circledast \tilde{B}^{(m)}\big)\big](z,\Omega), \tag{1}$$

where Ω is the frequency variable, $\tilde{B}^{(m)}(z,\Omega) = \mathscr{F}\{B^{(m)}(z,t)\}$ with $\mathscr{F}$ indicating the Fourier transform, α_0 is a propagation loss coefficient, β_1 is the reciprocal of the light group velocity $v_{\rm g}$ at a reference frequency ω_0, $\beta_k (k \geq 2)$ are dispersion coefficients associated with the Taylor expansion of the propagation constant $\beta(\omega)$ at ω_0, and γ is the nonlinear coefficient. Note that $\circledast$ denotes convolution and $\bar{\tilde{B}}(z,\Omega) = \tilde{B}^*(z,-\Omega)$, with * indicating the complex conjugate. In order to describe the evolution of an optical field in a microresonator, Supplementary Equation 1 must be complemented by the cavity boundary condition

$$\tilde{B}^{(m+1)}(0,\Omega) = \sqrt{1-\theta}e^{-i\delta_0}\tilde{B}^{(m)}(L,\Omega) + \sqrt{\theta}\tilde{E}_{\rm in}(\Omega), \tag{2}$$

where $\delta_0 = 2p\pi - \omega_0 t_{\rm R}$ is the linear phase detuning experienced by the carrier field with frequency ω_0 relative to the nearest (pth) cavity resonance, L and $t_{\rm R} = \beta_1 L$ are the cavity length and roundtrip time, respectively, θ is the input coupling coefficient, and $\tilde{E}_{\rm in}(\Omega)$ is the input pump field. It is convenient to introduce a change of variable $\tilde{B}^{(m)}(z,\Omega) = \tilde{E}^{(m)}(z,\Omega)e^{i\beta_1\Omega z}$ so that Supplementary Equations 1 and 2 are rewritten as

$$\frac{\partial \tilde{E}^{(m)}}{\partial z} = \bigg(-\frac{\alpha_0}{2} + i\sum_{k\geq 2}\frac{\beta_k}{k!}\Omega^k \bigg)\tilde{E}^{(m)}(z,\Omega) + i\gamma\big[\bar{\tilde{E}}^{(m)} \circledast \big(\tilde{E}^{(m)} \circledast \tilde{E}^{(m)}\big)\big](z,\Omega), \tag{3}$$

and

$$\tilde{E}^{(m+1)}(0,\Omega) = \sqrt{1-\theta}e^{-i(\delta_0-\beta_1\Omega L)}\tilde{E}^{(m)}(L,\Omega) + \sqrt{\theta}\tilde{E}_{\rm in}(\Omega). \tag{4}$$

This change of variable is equivalent to defining a shifting time reference frame, or $\tau = t - \beta_1 z$ such that τ and Ω form a pair of conjugate variables. Note that the mathematical form of the convolution term is preserved.

Let us now consider a specific pump field of the form $\tilde{E}_{\rm in}(\Omega) = A_{\rm in}[\delta(\Omega-\Omega_+)+\delta(\Omega-\Omega_-)]$, where $\Omega_+ = 2n\pi\cdot{\rm FSR}$ and $\Omega_- = -2n\pi\cdot{\rm FSR}$. Introducing this pump form into Supplementary Equation 4 and carrying out the usual procedures [2], we obtain the Lugiato-Lefever equation [3] for the dual-pump case

$$t_{\rm R}\frac{\partial E}{\partial t} = \bigg[-\frac{\alpha_0 L + \theta}{2} - i\delta_0 + iL\sum_{k\geq 2}\frac{\beta_k}{k!}\Big(i\frac{\partial}{\partial\tau}\Big)^k + i\gamma L|E|^2\bigg]E(t,\tau) + \sqrt{\theta}A_{\rm in}\big(e^{-i\Omega_+\tau} + e^{-i\Omega_-\tau}\big). \tag{5}$$

Here $t = mt_{\rm R}$ is a slow evolutionary time variable which keeps track of the number of roundtrips completed by the intracavity field. The pumps are symmetrically located with respect to the frequency ω_0 and each of them is an integer multiple (n) of FSR away from ω_0. Therefore, in the absence of dispersion, the two pumps and the degenerate signal are detuned to the same extent relative to their respective nearest resonances, that is, by δ_0.

It has been shown that Supplementary Equation 5 allows modeling of the dynamics of Kerr optical parametric oscillators under certain circumstances [4], however predicts for our current experimental system that there is no stable oscillation of the degenerate signal. A major limitation of Supplementary Equation 5 lies in the fact that the pumps and the signal are constrained to the same detuning δ_0. It completely rules out our ability to independently and individually tune the two pumps. To incorporate this additional experimental degree of freedom into our model, we are led to consider a more general case

$$\Omega_+ = 2n\pi\cdot{\rm FSR} + \delta\Omega_+ \tag{6}$$
$$\Omega_- = -2n\pi\cdot{\rm FSR} + \delta\Omega_-, \tag{7}$$

such that the pump becomes

$$\tilde{E}_{\rm in}(\Omega) = A_{\rm in}\big[\delta(\Omega - 2n\pi\cdot{\rm FSR} - \delta\Omega_+) + \delta(\Omega + 2n\pi\cdot{\rm FSR} - \delta\Omega_-)\big]. \tag{8}$$

Assuming the field $\tilde{E}^{(m)}$ in Supplementary Equation 3 varies slowly over a length-scale of one roundtrip, Supplementary Equation 3 can be integrated over the cavity length L by the first-order Euler's method. We substitute the result of the integration step into Supplementary Equation 4 and obtain

$$\begin{aligned}\tilde{E}^{(m+1)}(0,\Omega) =& \sqrt{1-\theta}e^{-i(\delta_0-\beta_1\Omega L)}\left[\left(1-\frac{\alpha_0 L}{2}+iL\sum_{k\geq 2}\frac{\beta_k}{k!}\Omega^k\right)\tilde{E}^{(m)}(0,\Omega)+i\gamma L\big[\bar{\tilde{E}}^{(m)}\circledast\big(\tilde{E}^{(m)}\circledast\tilde{E}^{(m)}\big)\big](0,\Omega)\right]\\ &+\sqrt{\theta}A_{\mathrm{in}}\big[\delta\big(\Omega-2n\pi\cdot\mathrm{FSR}-\delta\Omega_+\big)+\delta\big(\Omega+2n\pi\cdot\mathrm{FSR}-\delta\Omega_-\big)\big].\end{aligned} \tag{9}$$

Generally, $|\delta\Omega_+|\neq|\delta\Omega_-|$ and the spectral positions of the pumps are no longer symmetric about ω_0. The symmetry can be restored by introducing a shifted frequency Ω'

$$\Omega=\Omega'+\frac{\delta\Omega_+ +\delta\Omega_-}{2}=\Omega'+\epsilon, \tag{10}$$

such that the pump term in the shifted frequency is

$$\tilde{E}_{\mathrm{in}}(\Omega')=A_{\mathrm{in}}\left[\delta\left(\Omega'-2n\pi\cdot\mathrm{FSR}-\frac{\delta\Omega}{2}\right)+\delta\left(\Omega'+2n\pi\cdot\mathrm{FSR}+\frac{\delta\Omega}{2}\right)\right], \tag{11}$$

where $\delta\Omega=\delta\Omega_+-\delta\Omega_-$. Substituting the shifted frequency into Supplementary Equation 9 results in

$$\begin{aligned}\tilde{E}^{(m+1)}(0,\Omega') =& \sqrt{1-\theta}e^{-i(\delta_0-\epsilon\beta_1 L)}e^{i\beta_1\Omega' L}\left[\left(1-\frac{\alpha_0 L}{2}+iL\sum_{k\geq 2}\frac{\beta_k}{k!}(\Omega'+\epsilon)^k\right)\tilde{E}^{(m)}(0,\Omega')\right.\\ &\left.+i\gamma L\big[\bar{\tilde{E}}^{(m)}\circledast\big(\tilde{E}^{(m)}\circledast\tilde{E}^{(m)}\big)\big](0,\Omega')\right]\\ &+\sqrt{\theta}A_{\mathrm{in}}\big[\delta\big(\Omega'-2n\pi\cdot\mathrm{FSR}-\delta\Omega/2\big)+\delta\big(\Omega'+2n\pi\cdot\mathrm{FSR}+\delta\Omega/2\big)\big].\end{aligned} \tag{12}$$

Let us pay a close attention to the term $e^{i\beta_1\Omega' L}$. Since the pumps are no longer separated from each other by an integer number of the cavity FSR, we should be cautious with the definition of the frequency Ω'. More specifically, the frequency grid with a step size of FSR is not appropriate. Instead, the frequency axis should be discretized with adjacent points spaced by

$$\Delta\Omega=2\pi\Delta f=2\pi\cdot\mathrm{FSR}+\frac{\delta\Omega}{2n}. \tag{13}$$

As an example, we see that the qth frequency component from the central cavity mode will experience an additional frequency shift $[2q\pi\cdot\mathrm{FSR}+q\delta\Omega/(2n)]\beta_1 L=2q\pi+q\delta\Omega/(2n\cdot\mathrm{FSR})$, since $\beta_1 L=1/\mathrm{FSR}$. In other words, we have

$$\begin{aligned}e^{i\beta_1 q\Delta\Omega L} &= e^{i2\pi q}\cdot\exp\left\{\left(i\frac{q\delta\Omega}{2n\cdot\mathrm{FSR}}\right)\right\}\\ &=\exp\left\{\left(i\frac{q\delta\Omega}{2n\cdot\mathrm{FSR}}\right)\right\}.\end{aligned} \tag{14}$$

In view of the continuous variable Ω', Supplementary Equation 14 can equivalently be written as

$$e^{i\beta_1\Omega' L}=\exp\left\{\left(i\frac{2\pi}{\Delta\Omega}\Omega'\right)\right\}\cdot\exp\left\{\left(i\frac{\delta\Omega}{2n\cdot\mathrm{FSR}\cdot\Delta\Omega}\Omega'\right)\right\}, \tag{15}$$

such that Supplementary Equations 14 and 15 agree for $\Omega'=q\Delta\Omega$. Since $|\delta\Omega|\ll\mathrm{FSR}$, for sufficiently narrow-band structure, Supplementary Equation 15 can be expanded to first order. Substituting Supplementary Equation 15 into Supplementary Equation 12 and expanding all terms to first order lead to

$$\begin{aligned}\tilde{E}^{(m+1)}(0,\Omega') =& \left[1-\frac{\alpha_0 L+\theta}{2}-i\big(\delta_0-\epsilon\beta_1 L\big)+i\frac{\delta\Omega}{2n\cdot\mathrm{FSR}\cdot\Delta\Omega}\Omega'+iL\sum_{k\geq 2}\frac{\beta_k}{k!}\big(\Omega'+\epsilon\big)^k\right]\tilde{E}^{(m)}(0,\Omega')\\ &+i\gamma L\big[\bar{\tilde{E}}^{(m)}\circledast\big(\tilde{E}^{(m)}\circledast\tilde{E}^{(m)}\big)\big](0,\Omega')\\ &+\sqrt{\theta}A_{\mathrm{in}}\big[\delta\big(\Omega'-2n\pi\cdot\mathrm{FSR}-\delta\Omega/2\big)+\delta\big(\Omega'+2n\pi\cdot\mathrm{FSR}+\delta\Omega/2\big)\big].\end{aligned} \tag{16}$$

As in Supplementary Equation 5, we define the slow evolutionary time $t = mt_\mathrm{R}$ so that roundtrip-to-roundtrip variation of the field envelope can be approximated as a first-order derivative, that is,

$$\tilde{E}^{(m+1)}(0,\Omega') - \tilde{E}^{(m)}(0,\Omega') \approx t_\mathrm{R}\frac{\partial \tilde{E}(t=mt_\mathrm{R},\Omega')}{\partial t}. \tag{17}$$

Using Supplementary Equation 17, Supplementary Equation 16 is expressed as

$$\begin{aligned} t_\mathrm{R}\frac{\partial \tilde{E}(t,\Omega')}{\partial t} =& \Big[-\frac{\alpha_0 L+\theta}{2} - i\big(\delta_0 - \epsilon\beta_1 L\big) + i\frac{\delta\Omega}{2n\cdot\mathrm{FSR}\cdot\Delta\Omega}\Omega' + iL\sum_{k\geq 2}\frac{\beta_k}{k!}\big(\Omega'+\epsilon\big)^k\Big]\tilde{E}(t,\Omega') \\ &+ i\gamma L\big[\tilde{\bar{E}} \circledast \big(\tilde{E}\circledast\tilde{E}\big)\big](t,\Omega') \\ &+ \sqrt{\theta}A_\mathrm{in}\big[\delta\big(\Omega' - 2n\pi\cdot\mathrm{FSR} - \delta\Omega/2\big) + \delta\big(\Omega' + 2n\pi\cdot\mathrm{FSR}+\delta\Omega/2\big)\big]. \end{aligned} \tag{18}$$

Before transforming Supplementary Equation 18 back into the time domain, let us examine the dispersion term. Since the dispersion coefficients β_k were obtained from the Taylor expansion of the propagation constant $\beta(\omega)$ about the reference frequency ω_0, shifting the frequency axis (Supplementary Equation 10) results in a correction term to the term of each order. This can be seen more clearly by expanding the dispersion terms to the first order in ϵ

$$\sum_{k\geq 2}\frac{\beta_k}{k!}\big(\Omega'+\epsilon\big)^k \approx \beta_2\epsilon\Omega' + \sum_{k\geq 2}\frac{\beta_k + \beta_{k+1}\epsilon}{k!}\Omega'^k. \tag{19}$$

Substituting Supplementary Equation 19 into Supplementary Equation 18 and taking the inverse Fourier transform, we obtain the following mean-field equation

$$\begin{aligned} t_\mathrm{R}\frac{\partial E}{\partial t} =& \Big[-\frac{\alpha_0 L+\theta}{2} - i\big(\delta_0 - \epsilon t_\mathrm{R}\big) - \left(\frac{\delta\Omega}{2n\cdot\mathrm{FSR}\cdot\Delta\Omega} + \beta_2\epsilon L\right)\frac{\partial}{\partial\tau'} + iL\sum_{k\geq 2}\frac{\beta_k+\epsilon\beta_{k+1}}{k!}\left(i\frac{\partial}{\partial\tau'}\right)^k\Big]E(t,\tau') \\ &+ i\gamma L|E(t,\tau')|^2E(t,\tau') + \sqrt{\theta}A_\mathrm{in}\left[e^{-i(2n\pi\cdot\mathrm{FSR}+\delta\Omega/2)\tau'} + e^{i(2n\pi\cdot\mathrm{FSR}+\delta\Omega/2)\tau'}\right]. \end{aligned} \tag{20}$$

For most practical situations, $|\epsilon| \ll \mathrm{FSR}$ so that $|\epsilon\beta_{k+1}| \ll |\beta_k|$ and corrections to the dispersion terms can be neglected. Let us additionally define

$$\alpha = \frac{\alpha_0 L+\theta}{2} \tag{21}$$

$$\delta_0' = \delta_0 - \epsilon t_\mathrm{R} \tag{22}$$

$$\delta_1 = \frac{\delta\Omega}{2n\cdot\mathrm{FSR}\cdot\Delta\Omega} + \beta_2\epsilon L \tag{23}$$

$$\Omega_0 = 2n\pi\cdot\mathrm{FSR} + \frac{\delta\Omega}{2}. \tag{24}$$

Here, α is half the fractional roundtrip loss, δ_0' is the effective phase detuning, δ_1 is the mode-dependent detuning, and Ω_0 corresponds to the pump offset frequency from the degeneracy point. By substituting these new variables into Supplementary Equation 20, we obtain the following modified Lugiato-Lefever equation

$$t_\mathrm{R}\frac{\partial E}{\partial t} = \left[-\alpha - i\delta_0' - \delta_1\frac{\partial}{\partial\tau'} + iL\sum_{k\geq 2}\frac{\beta_k}{k!}\left(i\frac{\partial}{\partial\tau'}\right)^k + i\gamma L|E(t,\tau')|^2\right]E(t,\tau') + \sqrt{\theta}A_\mathrm{in}\big(e^{-i\Omega_0\tau'} + e^{i\Omega_0\tau'}\big). \tag{25}$$

Finally, we extend Supplementary Equation 25 to emulate a system of N coupled DOPO's. To this end, we introduce the complex coupling coefficient $\kappa_{jk} = |\kappa_{jk}|e^{i\phi_{jk}}$, which describes a linear coupling from the kth to j^th DOPO's with strength $|\kappa_{jk}|$ and phase ϕ_{jk}. The emulation of a system of N coupled DOPO's amounts to solving the following coupled Lugiato-Lefever equations

$$t_\mathrm{R}\frac{\partial E_j}{\partial t} = \left[-\alpha - i\delta_0' - \delta_1\frac{\partial}{\partial\tau'} + iL\sum_{k\geq 2}\frac{\beta_k}{k!}\left(i\frac{\partial}{\partial\tau'}\right)^k + i\gamma L|E_j(t,\tau')|^2\right]E_j(t,\tau') + \sqrt{\theta}A_\mathrm{in}\big(e^{-i\Omega_0\tau'} + e^{i\Omega_0\tau'}\big) + \sum_{k\neq j}\kappa_{jk}E_k(t,\tau'), \tag{26}$$

with $j, k = 1, 2, 3, ..., N$. Our experimental system of two DOPO's with unidirectional coupling can be simulated by setting $N = 2$, and $\kappa_{12} = 0$ and $\kappa_{21} = \kappa$ with κ being the experimentally measured coupling coefficient. The coupling phases of 0 and π correspond to the cases of in-phase and out-of-phase couplings, respectively. Under these conditions, dropping the primes in Supplementary Equation 26 leads to the coupled equations in the main text.

For our simulations, we use the following parameters: $\alpha = \theta = 0.0019$, $t_R = 5$ ps, $L = 340$ µm, $\gamma = 1.23$ W^{-1}m^{-1}, $\beta_2 = 57.4$ ps^2/km, $\beta_3 = -1.2$ ps^3/km, $\beta_4 = 0.009$ ps^4/km, $A_{in} = 0.19$ W$^{1/2}$, $\Omega_0 = 2\pi \times 1.004$ THz, and $|\kappa| = 0.048 \cdot \theta \approx 9.1 \times 10^{-5}$. Since the detuning parameters δ_0 and δ_1 are difficult to measure experimentally, we assume values which reasonably reproduce the experimental results. For the simulation results presented as Fig. 5 in the main text, we use $\delta_0' = 0.0038$ and $\delta_1 = 7.5 \times 10^{-16}$ s. The dispersion coefficients β_k and the nonlinear coefficient γ are calculated by performing a finite element simulation based on the commercial COMSOL Multiphysics package. All other parameters are experimentally measured or estimated.

Supplementary Note 5 - Characterization of DOPO with low pump power

We have characterized a single SiN microresonator to determine the oscillation threshold of the system. The SiN microresonator has a cross section of 730×1050 nm and an FSR of 500 GHz. The quality factor is 1.6 million. Supplementary Figure 7 shows the generated spectra for three different combined pump powers. We achieve oscillation with combined pump powers as low as 50 mW. The data point is included in Fig. 6 in the main text.

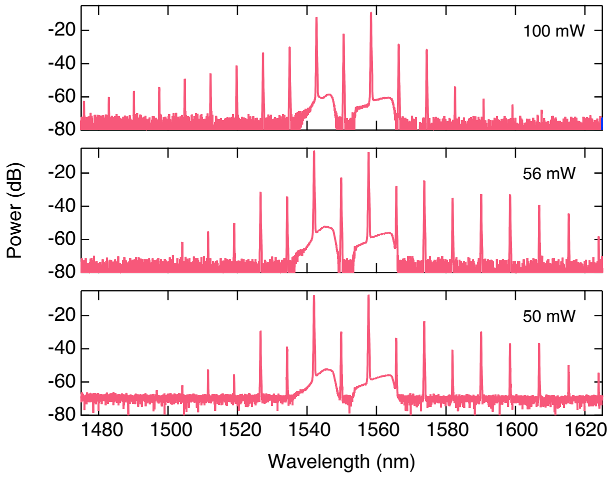

Supplementary Figure 7: **Low power DOPO generation.** The generated DOPO spectra is shown for three different pump powers (100 mW, 56 mW, and 50 mW from top to bottom). The SiN microresonator has a 500-GHz FSR with a cross section of 730×1050 nm.

Supplementary Note 6 - Characterization of system scalability

In order to allow for simultaneous parametric oscillation, the resonances for $DOPO_1$ and $DOPO_2$ must be matched. To characterize the deviation of the resonances for the two microresonators in our coupled-DOPO system, we have performed measurements of the absolute wavelength of the lower wavelength pump for the 10 devices that are on one chip. Supplementary Figure 8 shows the low wavelength pump resonance for $DOPO_1$ and $DOPO_2$ (top) and the difference between the two resonance frequencies (bottom). Here, each die is 1×1 mm so the 10 devices span over 1 cm. We calculate an average difference between the two frequencies of 4.68 GHz. We attribute the variation in the resonance frequencies between the two microresonators within the same die to the resolution of electron beam lithography. Based on numerical modeling of our waveguide geometry, a 1-nm variation in the microresonator width corresponds to a resonance shift of 13 GHz. We see that all but one die

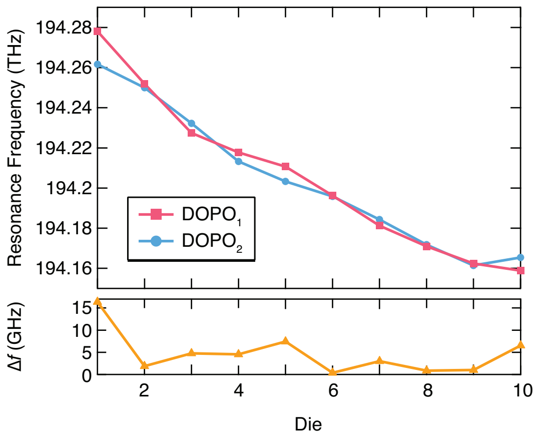

Supplementary Figure 8: **Resonance characterization of coupled-DOPO system.** (Top): Measured resonance wavelength for the low wavelength pump for each die on the coupled-DOPO device. There are 10 dies on a chip with each die having a 1×1 mm footprint. (Bottom): Absolute difference in resonance wavelength Δf between the two DOPO's.

has a frequency difference well within this range, confirming that we have variation within 1-nm. In addition to the variation within a single die, we also observe a decrease in resonance frequency of 120 GHz over the 10 dies. We believe that this is due to the nonuniformity of the SiN film thickness due to the deposition process. There are several ways to address this issue. First, our silicon nitride films are deposited in a two-step process, between the two steps, we can twist the wafer orientation and change the wafer position to compensate the film thickness variations [5]. Second, a post chemical mechanical planarization (CMP) process can be used to reduce the film thickness variations. Many parameters can be tuned in the CMP machine such as backside pressure, rotation speed and load pressure, down load pressure, such that different film removal rates can be achieved at different locations. Previous demonstrations have shown reduction of the root-mean-squared roughness of the top surface of SiN from 0.38 nm to 0.08 nm [6]. Third, a wafer map can be measured using measurement tools such as Filmetrics, which can provide nm or even sub-nm film thickness accuracy. With the wafer map, the devices' widths can be designed to compensate the relative height difference across the wafer. With this accuracy in film thickness the deviation in FSR will be largely due to the electron beam resolution. Previous work has shown that it is possible to fabricate structure below 1 nm in size [7], which is consistent with our characterization based on our measured resonance variations.

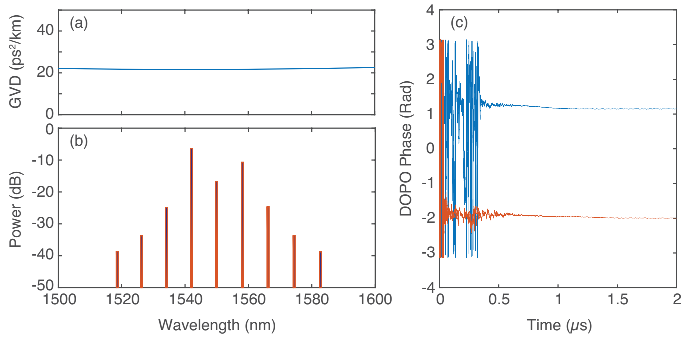

Supplementary Figure 9: **DOPO dynamics for 10 million intrinsic Q.** (a) Group-velocity dispersion (GVD) for 730×3000 nm cross section microresonator with 500-GHz FSR. (b) Generated DOPO spectrum. The combined pump power is 1 mW. The two highest peaks correspond to the pump. (c) Time dynamics of the DOPO for two different initiations. The plot shows the two different final phase states offset by π.

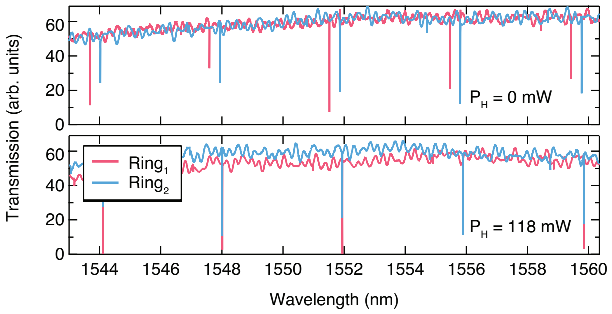

Supplementary Figure 10: **Transmission measurement of microresonators.** The transmission through the two microresonators ($DOPO_1$ and $DOPO_2$ are characterized for two different electrical powers applied to the heater.

Next, we calculate the expected free-spectral range (FSR) mismatch based on a 1 nm difference in microresonator width. The resonance frequency satisfied the condition $2\pi m = n(\omega)\frac{\omega}{c}L$. We assume that $DOPO_2$ is tuned thermally to match $DOPO_1$. The FSR's of $DOPO_1$ and $DOPO_2$ can be expressed as $\text{FSR}_1 = \frac{c}{[n_1(\omega)+\omega\frac{dn_1}{d\omega}]L}$ and $\text{FSR}_2 = \frac{c}{[n_2(\omega)+\omega\frac{dn_2}{d\omega}]L}$, respectively, where n is the effective index, L is the circumference of the resonator, and Δn is the thermal index change. In order for the two resonances to match at $\omega = \omega_1$, the condition $n_1(\omega_1) = n_2(\omega_1) + \Delta n$ must be satisfied. Thus, the FSR equations at $\omega = \omega_1$ simplify to $\text{FSR}_1 = \frac{c}{[n_1(\omega_1)+\omega_1\frac{dn_1}{d\omega}]L}$ and $\text{FSR}_2 = \frac{c}{[n_1(\omega_1)+\omega_1\frac{dn_2}{d\omega}]L}$. We use a finite element mode solver to model the wavelength dependent effective index of our device. For our coupled-DOPO device in the main text, the waveguide cross section is 730×1050 nm. For a 1 nm change in waveguide width, we calculate a 43.5 MHz change in FSR. This FSR difference is sufficient for our current system which has a resonance linewidth of 300 MHz. However, for higher Q-factor microresonators, the FSR variation must be significantly reduced. To achieve this, we consider a wider width microresonator with a cross section of 730×3000 nm. Remarkably, the FSR difference is significantly reduced to 2.9 MHz for a 1-nm change in width. This allows for alignment of the DOPO's within the 19 MHz linewidth for a $Q = 10^7$. We have verified through numerical modeling that the system indeed oscillates with 1 mW of pump power (Supplementary Figure 9). The GVD still satisfies the conditions required for degenerate parametric oscillation [1], and we observe two distinct DOPO phases for different initiations that are offset by π. This approach provides a path towards scaling our system to a large number of coupled-DOPO's.

In addition, we characterize the thermal tuning properties of our microresonators. Here, we use two separate microresonators, Ring_1 and Ring_2, with similar FSR's and characterize the frequency shift using integrated heaters for a resonance near 1560 nm. To better characterize the thermal frequency shift, we choose two microresonators with a relatively large resonance offset. Supplementary Figure 10 shows the transmission measurement of the two rings. We characterize the resonance frequency shift Δf_m as the electrical power to the integrated heater for Ring_1 is changed. When the heater is turned off, the difference in resonance wavelengths near 1560 nm is 0.4 nm. We measure the transmission for several electrical power values and extract a thermal frequency coefficient of 0.42 GHz/mW. For the DOPO system described in the main text, we apply 10.9 mW of electrical power to the heater. Based on this coefficient, the frequency shift $\Delta f_m = 4.6$ GHz, which agrees well with the frequency difference shown in Supplementary Figure 8.

Supplementary Note 7 - Numerical demonstration of an integrated photonic Ising machine

The aim of this section is to establish that our proposed system indeed minimizes the Ising Hamiltonian and behaves as a photonic Ising machine. We employ the coupled Lugiato-Lefever equations (Supplementary Equation 26) to tackle the Max-Cut problem of an undirected graph with 100 nodes. The graph we consider is the configuration known as Möbius ladder, the Max-Cut problem of which has been used as a bench-mark problem for evaluating the performance of physical photonic Ising machines [8]. We encode this problem by enforcing $\kappa_{j,j+1} = \kappa_{j,j-1} = \kappa_{j,j+\frac{N}{2}} = -|\kappa|$, where $N = 100$, and $|\kappa| = 0.048 \cdot \theta \approx 9.1 \times 10^{-5}$ is the strength of the coupling used in our experiment. The periodic boundary condition $E_{N+1} = E_1$ and $E_0 = E_N$ is also enforced. The lack of directions

in the graph is satisfied by the symmetry constraint $\kappa_{jk} = \kappa_{kj}$. By numerically solving the 100 coupled Lugiato-Lefever equations and tracking the evolution of the signal phases of all 100 DOPO's, we indeed confirm that our system attempts to minimize the Ising Hamiltonian. Typical evolutions of the signal powers and phases are shown in Supplementary Figure 11(a) and (b). In addition, Supplementary Figure 11(c) shows the evolution of the associated Ising Hamiltonian, which clearly reveals that the system finds the ground state with the associated minimum energy of -146 (see the argument below). In this specific trial, we use $\delta_0' = 0.0031$ and $\delta_1 = 7.5 \times 10^{-16}$ s. By ramping the detuning at a rate of $10^{-6}\alpha$ per roundtrip (see Supplementary Figure 12), we achieve an annealing time $T_{\text{ann}} = 0.2$ µs with a 35 % ground state success probability. We calculate a time to solution $T_{\text{sol}} = \frac{\log(0.01)}{\log(1-P)} = 2.1$ µs [9, 10]. This indicates that the spatial-multiplexed DOPO system potentially offers substantial acceleration as compared to the fiber-based DOPO system ($T_{\text{sol}} = 2.3$ ms) [10] and the photonic recurrent Ising sampler ($T_{\text{sol}} < 1$ ms) [11] for solving cubic Max-Cut problems with $N = 100$, and compares favorably to the state-of-the-art digital and electronic systems [12].

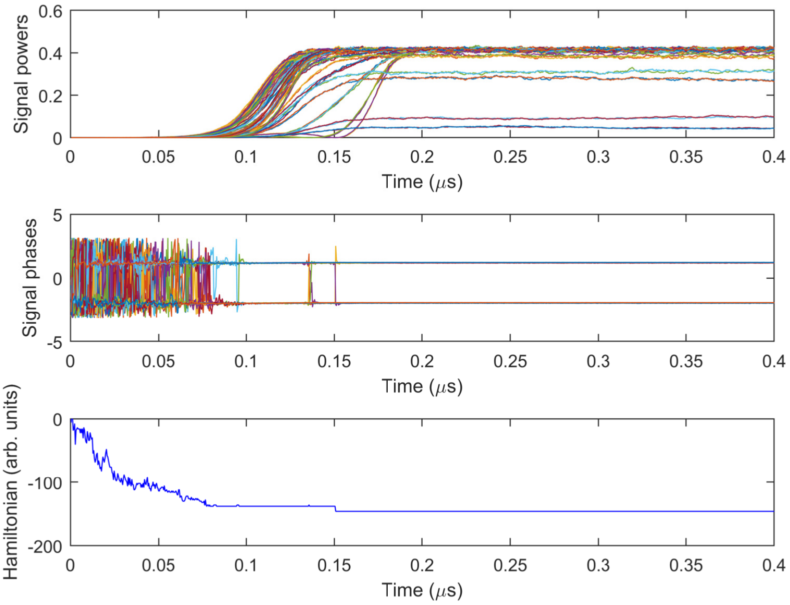

Supplementary Figure 11: **Simulated evolution of the 100 coupled DOPO's.** Evolutions of the (a) signal powers, (b) signal phases, and (c) the associated Ising Hamiltonian.

The highly symmetric nature of the Möbius ladder configuration enables us to deduce the maximum cut as follows. Let us first consider a one-dimensional ring of $N = 2n$ (n is an arbitrary natural number such that N is an even number) nodes with the nearest-neighbor coupling of equal weighting. The ith node can have either $s_i = +1$ (spin up) or $s_i = -1$ (spin down). This ring configuration satisfies the periodic boundary condition $s_{N+1} = s_1$. The Max-Cut problem of this graph can be implemented by assigning $J_{i,i+1} = J_{i+1,i} = -1$, and 0 otherwise, with the resulting Hamiltonian $H = \sum_{i=1}^{N} s_i s_{i+1}$. It is easy to see that the energy is minimized when we assign spins of alternating signs along the ring. The associated minimum energy is $H_{\text{min}} = -N$. Note that there is an inherent degeneracy arising from an arbitrary choice of the spin in the first randomly chosen node. We ignore this degeneracy. The one-dimensional ring can be extended to the corresponding Möbius-ladder configuration by adding an edge between each node and the node half-way across the ring, that is, $J_{i,i+N/2} = -1$, for all $i = 1, 2, ..., N$. Now two possible scenarios arise and we must consider them separately.
(1) If $N = 2n$ is not a multiple of 4, the same spin configuration as that of the corresponding ring minimizes the system energy with the new minimum energy of $H_{\text{min}} = -3N/2$. This is because $N/2$ is an odd number so that $s_i s_{i+N/2} = (-1)^{N/2}(s_i)^2 = -1$. Therefore, each new connection reduces energy by one unit.
(2) If $N = 2n$ is a multiple of 4, the spin configuration of the ring is no longer the optimal solution. This is

because now $N/2$ is an even number such that $s_i s_{i+N/2} = +1$. Moreover, this penalty in energy gets multiplied by $N/2$ which is the number of cross-connecting edges. We therefore search for a more optimal configuration by introducing at least one domain wall. We readily see that the configuration with a pair of domain walls that are separated by $N/2$ nodes is the most energy-favorable solution. These domain walls only add a fixed energy penalty of $+2$, independent of N, while all the conflicts are resolved for the cross-connections. Therefore, the minimum energy is $H_{\text{min}} = -\left(\frac{3N}{2} - 2\right) + 2 = -\frac{3N}{2} + 4$.
Our example simulation case of $N = 100$ nodes is an element of the second scenario and we expect the minimum energy to be $H_{\text{min}} = -146$, which is consistent with what our simulation predicts [see Supplementary Figure 11(c)] and with [8]. We also see that the solution is degenerate with a multiplicity of $N/2$, due to the underlying symmetry of the graph itself and the absence of external magnetic field. The absolute location of the domain wall pair does not matter, as long as they are on opposite ends. This degeneracy is also demonstrated in our simulation which shows no preference for any one specific configuration of all possible optimal solutions.

Supplementary Note 8 - Theoretical analysis of DOPO network

In this section, we theoretically investigate the operation of a large-scale network of Kerr DOPO's as a photonic Ising machine. We first treat a single isolated DOPO. Although we have derived a modified Lugiato-Lefever equation (Supplementary Equation 25) to model our DOPO, it is not easy to reveal the behavior of a DOPO directly from Supplementary Equation 25 in its current form. We therefore simplify our analysis by only considering the evolutions of the two pumps and the frequency-degenerate signal. We also limit ourselves to the degenerate detuning case by setting $\delta_1 = 0$, and only retain the second-order dispersion coefficient, *i.e.* $\beta_k = 0$ for $k \geq 3$. We first normalize Supplementary Equation 25 according to the conventions presented in [13], and introduce the ansatz $E(t,\tau) = E_{\text{S}}(t) + E_+(t)e^{-i\Omega_0\tau} + E_-(t)e^{i\Omega_0\tau}$ into the normalized Lugiato-Lefever equation, where E_{S} and $E_\pm$ are slowly varying Fourier amplitudes of the signal and two pumps, respectively. After simplification, we obtain the following set of coupled ordinary differential equations

$$\frac{dE_+}{dt} = \big[-1 - i\big(\Delta - d_2\Omega_0^2 - |E_+|^2 - 2|E_-|^2 - 2|E_{\text{S}}|^2\big)\big]E_+ + iE_{\text{S}}^2E_-^* + S \tag{27}$$
$$\frac{dE_-}{dt} = \big[-1 - i\big(\Delta - d_2\Omega_0^2 - 2|E_+|^2 - |E_-|^2 - 2|E_{\text{S}}|^2\big)\big]E_- + iE_{\text{S}}^2E_+^* + S \tag{28}$$
$$\frac{dE_{\text{S}}}{dt} = \big[-1 - i\big(\Delta - 2|E_+|^2 - 2|E_-|^2 - |E_{\text{S}}|^2\big)\big]E_{\text{S}} + 2iE_+E_-E_{\text{S}}^*, \tag{29}$$

where S, Δ and d_2 are the dimensionless pump strength, detuning and GVD coefficient, respectively. In the small signal limit, we can find the steady-state pump fields by solving Supplementary Equations 27 and 28. Let us assume we have found the steady-state pump fields $E_+ = E_- = \sqrt{P}e^{i\phi_{\text{p}}}$, with power P and phase ϕ_{p}. Next, we separate Supplementary Equation 29 into its real and imaginary parts $E_{\text{S}} = s_{\text{R}} + is_{\text{I}}$ and obtain

$$\frac{d}{dt}\begin{pmatrix} s_{\text{R}} \\ s_{\text{I}} \end{pmatrix} = \begin{pmatrix} -1 - 2P\sin(2\phi_{\text{p}}) & 4P - \Delta - P\cos(2\phi_{\text{p}}) \\ -\Delta + 4P + P\cos(2\phi_{\text{p}}) & -1 + 2P\sin(2\phi_{\text{p}}) \end{pmatrix}\begin{pmatrix} s_{\text{R}} \\ s_{\text{I}} \end{pmatrix}. \tag{30}$$

The eigenvalues of the system matrix above can be found to be

$$\lambda_\pm = -1 \pm \sqrt{(6P - \Delta)(\Delta - 2P)}, \tag{31}$$

with the corresponding eigenvectors

$$\mathbf{v}_\pm = \begin{pmatrix} \frac{\Delta - 2P - 4P\sin^2(\phi_{\text{p}})}{2P\sin(2\phi_{\text{p}}) \pm 1} \\ \\ 1 \end{pmatrix}. \tag{32}$$

The signal is amplified when the eigenvalue λ_+ exceeds 0, that is, when the gain exceeds the normalized loss of -1. Thus, there are only two possible directions, $\mathbf{v}_+$ and $-\mathbf{v}_+$, along which the signal can be amplified. This explains the origin of the binary phase operation of our DOPO. This can also be seen by introducing the transformation $E_{\text{S}} \to E_{\text{S}}e^{i\pi}$ into Supplementary Equation 29 and noting that the equation remains invariant, which implies that if E_{S} is a non-trivial steady-state solution of Supplementary Equation 29, $-E_{\text{S}}$ is also a solution.

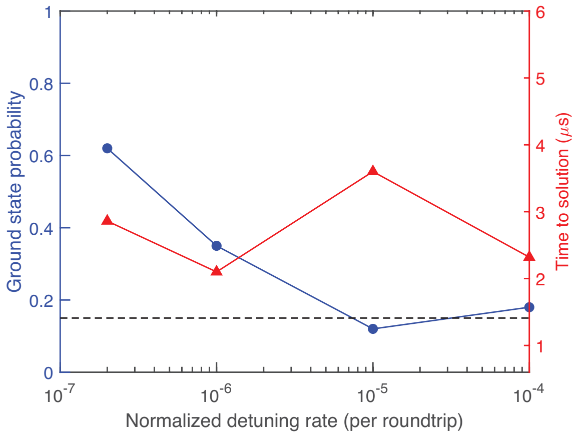

Supplementary Figure 12: **The success probability of global minimum search for various rates of detuning ramping.** The horizontal black dashed line corresponds to the scenario where the detuning is fixed at the final value throughout the simulation.

Having rigorously established the binary phase operation of our DOPO, we next look at a network of coupled DOPOs with arbitrary connections. We introduce the index i to denote the ith DOPO. We assume that the coupling strength is uniform throughout the network, which is consistent with our simulation of the unweighted max-cut problem presented in the previous section. We also neglect the coupling of the pumps which are assumed to be negligible. In this case, all the DOPO's have identical pumps. Then, we have

$$\frac{dE_{\text{S},i}}{dt} = \big[-1 - i\big(\Delta - 4P\big)\big]E_{\text{S},i} + 2i(Pe^{2i\phi_{\text{P}}})E^*_{\text{S},i} + |\kappa| \sum_{k\neq i} e^{i\psi_{ik}} E_{\text{S},k}. \tag{33}$$

At threshold, we can also assume that all DOPO's have identical magnitudes and are only allowed to vary in phase by π. It can be readily seen that the DOPO's will orient their spins such that the overall system loss is minimized. In the ideal situation, the coupling phases will be either 0 or π, in which case the loss is minimized if $e^{i\psi_{ik}}E_{\text{S},k} = E_{\text{S},i}$ and the equation is reduced to

$$\frac{dE_{\text{S},i}}{dt} = \big[-\big(1 - N|\kappa|\big) - i\big(\Delta - 4P\big)\big]E_{\text{S},i} + 2i(Pe^{2i\phi_{\text{P}}})E^*_{\text{S},i}. \tag{34}$$

Note how the loss has been reduced by $N|\kappa|$, where N is the number of DOPO's that are connected to the ith DOPO. We immediately deduce two decisive factors which influence the ground state probability: (1) the rate at which the pumps are tuned into resonance, and (2) the coupling strength. In particular, the former is tied to the fact that the system must be allowed to reside in the ideal detuning range where only the ground state experiences gain, before it crosses the oscillation threshold for the next excited state.

Finally, the above analysis can be generalized to non-ideal situations where the coupling phases are not exactly 0 or π. In this case, the effective loss becomes $1 - |\kappa| \sum_{k\neq i} \cos(\psi_{ik})$ and the system becomes less effective in distinguishing the ground state from the next excited state. In particular, when $\psi_{ik} = \pi/2 + m\pi$, where m is an arbitrary integer, the DOPO network will operate essentially in a random manner, which is consistent with our experimental result of the frustrated case.

We numerically verify the significance of the rate at which the pumps are tuned into resonance from below the oscillation threshold of the DOPO network. The Lugiato-Lefever equations (Supplementary Equation 26) are employed in this investigation and we defer the further discussion of Supplementary Equations 27-29 to a later study. We perform our simulation as follows. We initially set the pump detunings from the resonances ($\delta'_{0,\text{initial}} = -1.6 \cdot \alpha$) such that the system is below the threshold for the collective oscillation. While numerically integrating the Lugiato-Lefever equations, we ramp up the detunings at a constant rate to a final value ($\delta'_{0,\text{final}} = -1.45 \cdot \alpha$) slightly above the threshold. We investigate several different ramping speeds. For each ramping speed, we perform 100 independent trials in order to estimate the success probability of the global minimum search. The result is shown in Supplementary Figure 12, where the detuning rate (per roundtrip) is normalized to the total cavity loss

α. For comparison, we have run 100 independent trials for the scenario in which the detuning is fixed at the final value (*i.e.* $\delta'_0 = \delta'_{0,\text{final}}$) throughout the simulation, the result of which is marked as a horizontal black dashed line in the same figure. The ramping speed is clearly a decisive factor in the overall performance of our Ising machine. In particular, we observe from the direct comparison with the fixed detuning case that the system must be tuned at a sufficiently slow rate in order to improve the performance. Also on the same axis, we superimpose a plot of time to solution for the different detuning rates. As expected, for a problem of fixed geometry and size, the time to solution remains relatively constant regardless of the ramping speed. Slight fluctuations are likely to be statistical in nature (see, for example, that the ground state probability is slightly lower for a rate of 10^{-5} than 10^{-4}) and should diminish with an increased number of samples. While in principle, it is possible to simulate at an even slower rate, the direct integration of the Lugiato-Lefever equations is computationally too wasteful to pursue such a study. We will report elsewhere a more comprehensive investigation of our Ising machine based on a more efficient truncated Fourier-mode analysis which was briefly introduced above (Supplementary Equations 27-29).

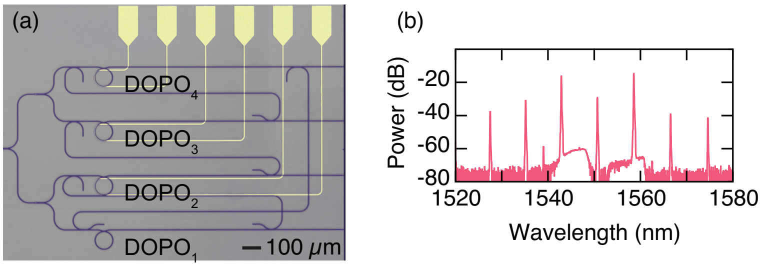

Supplementary Figure 13: **4-DOPO system.** (a) Microscope image of 4-DOPO device. MMI splitter is used to router the pumps to four SiN microresonators ($DOPO_1$ to $DOPO_4$ from bottom to top). Coupling waveguides allow for adjacent coupling between the DOPO's. To couple $DOPO_4$ to $DOPO_1$ the coupling waveguide intersects several waveguides. (b) The measured optical spectrum generated in $DOPO_3$.

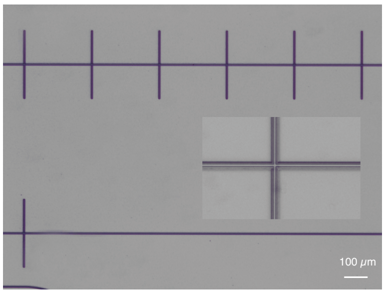

Supplementary Figure 14: **Microscope image of waveguides with crossings.** Two different devices are characterized, one with one crossing and the other with 6 crossings. Inset shows the zoom-in of the crossing region. We extract a loss of 0.45 dB per intersection.

Supplementary Note 9 - Characterization of 4-DOPO system

We preform measurements on a 4-DOPO system as shown in Supplementary Figure 13(a). MMI splitters in series are used to route the pumps to four SiN microresonators. Unidirectional coupling is implemented between each adjacent DOPO by using a coupling waveguide between the bus waveguides. To maintain a single plane, the coupling waveguide between $DOPO_4$ and $DOPO_1$ crosses the bus waveguide for $DOPO_2$, $DOPO_3$, and the coupling waveguide between $DOPO_1$ and $DOPO_2$. Supplementary Figure 13(b) shows the generated spectrum for one of the DOPO's.

Supplementary Note 10 - Characterization of crossing loss

As the number of microresonators increase in our system, in order to allow for coupling between the DOPO's in a single device layer, we require crossings between waveguides. To verify that we have low loss for the intersections between waveguides, we design devices with different number of crossings to characterize the loss (Supplementary Figure 14). We measure 0.45 dB loss per crossing. This can be further optimized by using a taper design at the crossing point.

Supplementary References

[1] Okawachi, Y. *et al.* Dual-pumped degenerate Kerr oscillator in a silicon nitride microresonator. *Opt. Lett.* **40**, 5267 (2015).

[2] Haelterman, M., Trillo, S. & Wabnitz, S. Dissipative modulation instability in a nonlinear dispersive ring cavity. *Optics Communications* **91**, 401 – 407 (1992).

[3] Lugiato, L. A. & Lefever, R. Spatial dissipative structures in passive optical systems. *Phys. Rev. Lett.* **58**, 2209 (1987).

[4] Jang, J. K., Okawachi, Y. & Gaeta, A. L. Dynamics of coupled microresonator-based degenerate optical parametric oscillators. In *Conference on Lasers and Electro-Optics*, FTh1E.4 (Optical Society of America, 2018).

[5] Dirani, H. E. *et al.* Ultralow-loss tightly confining Si_3N_4 waveguides and high-q microresonators. *Opt. Express* **27**, 30726–30740 (2019).

[6] Ji, X. *et al.* Ultra-low-loss on-chip resonators with sub-milliwatt parametric oscillation threshold. *Optica* **4**, 619 (2017).

[7] Broers, A. N. Resolution limits for electron-beam lithography. *IBM Journal of Research and Development* **32**, 502–513 (1988).

[8] McMahon, P. L. *et al.* A fully programmable 100-spin coherent Ising machine with all-to-all connections. *Science* **354**, 614 (2016).

[9] Aramon, M. *et al.* Physics-inspired optimization for quadratic unconstrained problems using a digital annealer. *Frontiers in Physics* **7**, 48 (2019).

[10] Hamerly, R. *et al.* Experimental investigation of performance differences between coherent Ising machines and a quantum annealer. *Science Advances* **5**, eaau0823 (2019).

[11] Roques-Carmes, C. *et al.* Heuristic recurrent algorithms for photonic Ising machines. *Nature Communications* **11**, 249 (2020).

[12] Cai, F. *et al.* Harnessing intrinsic noise in memristor Hopfield neural networks for combinatorial optimization. preprint at https://arxiv.org/abs/1903.11194 (2019).

[13] Leo, F. *et al.* Temporal cavity solitons in one-dimensional Kerr media as bits in an all-optical buffer. *Nature Photonics* **4**, 471–476 (2010).
